# Matrine effectively inhibits the proliferation of breast cancer cells through a mechanism related to the NF-κB signaling pathway

**DOI:** 10.3892/ol.2013.1399

**Published:** 2013-06-14

**Authors:** HONGMIN SHAO, BAOWEN YANG, RONGRONG HU, YINGYING WANG

**Affiliations:** Department of Oncology, Hospital of Traditional Chinese Medicine, Yantai, Shandong 264000, P.R. China

**Keywords:** matrine, breast cancer, NF-κB, IKKβ

## Abstract

Matrine is an alkaloid isolated from *Sophora flavescens*. The present study aimed to determine whether matrine effectively inhibits the proliferation of breast cancer cells, and the underlying mechanism(s) of its antitumor function. The effects of matrine on the cell viability of ER-positive MCF7 cells, HER2-positive BT-474 cells and highly metastatic MDA-MB-231 cells were measured using MTT and apoptosis assays. Western blot analysis was performed to investigate the expression levels of the inhibitor of κB (IκB) kinase β (IKKβ) in cells treated with or without matrine. It was observed that the matrine treatment resulted in the death of the three types of cancer cells, but significantly less toxicity was observed in the control cancer cells. The experimental results also suggested that the antitumor effects of matrine on breast cancer cells may be associated with the downregulation of IKKβ expression by matrine, as indicated by the western blot analysis results. The present results suggested that matrine may be used as an effective drug candidate for treating breast cancers in the future, following further research.

## Introduction

Matrine is an alkaloid isolated from *Sophora flavescens*, which has multiple functions, including acting as an analgesic reagent or against infection by pathogenic microorganisms (1–6). Matrine may also be used as an antioxidant that acts by promoting cell metabolism and regulating immune activities (7–10). It has been demonstrated that matrine has therapeutic effects on a variety of solid tumors, including breast, lung, stomach, esophageal, colorectal, cervical and ovarian cancer, as well as malignant lymphoma (11–13). However, the molecular mechanism underlying the antitumor function of matrine remains unclear.

The cellular nuclear factor-κB (NF-κB) signaling pathway is essential in various cellular processes, including cell survival, proliferation and apoptosis, which are important for the development of various types of human cancers (14–16). Under unstimulated conditions, the human NF-κB transcription factors are bound by the inhibitor of κB (IκB) proteins (17). However, pathological stimuli or environmental factors may result in the activation of NF-κB. Activation of IκB kinases (IKKs), including IKKα and IKKβ, results in the phosphorylation of IκB and its subsequent ubiquitin-dependent degradation by the proteasomal pathway (18,19). The released NF-κB transcription factors then translocate to the nucleus to regulate the expression of genes encoding cytokines, cytokine receptors and apoptotic regulators (20,21).

IKKβ has been demonstrated to be involved in development of numerous types of human tumors (22,23). In the present study, the effects of matrine treatment on multiple breast cancer cell lines, including ER-positive MCF7 cells, HER2-positive BT-474 cells and the highly metastatic MDA-MB-231 cell line, were determined. It was observed that the matrine treatment resulted in the death of the three types of cancer cells, but significantly less toxicity was observed in the control cancer cells. Our results suggest that matrine may be an effective approach for treating breast cancer in the future upon further research.

## Materials and methods

### Reagents and cell lines

Matrine (chemical formula, C_15_H_24_N_2_O; molecular weight, 248.36) was purchased from Sigma (cat. no. M5319-100MG; St. Louis, MO, USA). Matrine was dissolved in RPMI-1640 medium for use (1–4 mM). Three breast cancer cell lines, ER-positive MCF7 cells, HER2-positive BT-474 cells and the highly metastatic MDA-MB-231 cell line, were provided by the Department of Oncology, Hospital of Traditional Chinese Medicine (Yantai, China). MCF-7 cells, BT-474 cells and MDA-MB-231 cells were cultured in α-MEM, RPMI and DMEM (Sigma-Aldrich Co., Ltd., Irvine, CA, USA), respectively. The cells were cultured at 37°C with 5% CO_2_ and 100% humidity. The medium was supplemented with 10% fetal bovine serum (FBS; Hyclone, Logan, UT, USA), 100 U/ml penicillin and 100 *μ*g/ml streptomycin.

### Cell treatment and 3-(4,5-dimethylthiazol-2-yl)-2,5-diphenyltetrazolium bromide (MTT) assay

Briefly, cells were seeded into six-well plates in medium at a density of 1×10^5^ cells/well and cultured for 24 h. The cells were then treated with matrine (0, 1, 2 and 3 mM). The untreated cells were used as negative controls. Upon termination of each experiment (after 48 h), the cells were incubated with 0.5 mg/ml MTT for 4 h, according to the manufacturer’s instructions. The viability of the treated cells was expressed as relative to that of the control cells (relative viability).

### Apoptosis assay

Cells, at a density of 1×10^5^ cells/well, were cultured in six-well plates in medium supplemented with 10% calf serum for 24 h, followed by the addition of matrine (0, 1, 2 and 3 mM) or fresh medium (the untreated control). After 48 h, the cells were pelleted by centrifugation, washed twice with phosphate-buffered saline (PBS), fixed by incubation in 4% paraformaldehyde for 30 min at room temperature, and washed again with PBS to remove the fixative. The fixed cells were resuspended in PBS containing Hoescht 33258 (5 *μ*g/ml), followed by an incubation at room temperature for 15 min in the dark. The cells were placed on glass slides and examined for those with apoptotic morphology (nuclear condensation and chromatin fragmentation) via fluorescence microscopy (Labomed LX 400 fluorescence microscope; Labomed Inc., Culver City, CA, USA). To quantify the apoptosis, 250 nuclei from random microscopic fields were analyzed. Data are presented as the mean percentages of apoptotic cells.

### Western blot analysis

Cells, at a density of 1×10^5^ cells/well, were cultured in six-well plates in medium supplemented with 10% calf serum for 24 h, followed by the addition of matrine (0, 1, 2 and 3 mM) or fresh medium (the untreated control). After 48 h, the cells were pelleted by centrifugation and washed twice with PBS. Total proteins were harvested from the cells, then separated on 10% sodium dodecyl sulfate-polyacrylamide gel electrophoresis (SDS-PAGE) gels and subjected to immunoblot analyses. The primary antibodies against IKKβ (∼90 kDa) and β-actin were purchased from Santa Cruz Biotechnology, Inc. (Santa Cruz, CA, USA; anti-IKKβ, cat. no. sc-8014, 1:200; anti-β-actin, cat. no. sc-130301, 1:10,000). The secondary antibody used in the present study was goat anti-mouse IgG-horseradish peroxidase (HRP) (cat. no. sc-2005, 1:10,000; Santa Cruz Biotechnology, Inc.). Bound antibodies were detected using the ECL system (Cat No. 32134; Pierce Biotechnology, Inc., Rockford, IL, USA). The immunoblot experiments were repeated at least three times. The mean normalized optical densities (ODs) of the IKKβ protein bands relative to the ODs of the β-actin bands from the same condition were calculated.

### Statistical analysis

The experimental data are presented as the mean ± standard error (SEM). Statistical software (SPSS 12.0; SPSS, Inc., Chicago, IL, USA) was used to perform independent sample t-tests, followed by one-way analysis of variance. P<0.05 was considered to indicate a statistically significant difference.

## Results

### Matrine is toxic to breast cancer cell lines

**Figure 1.**
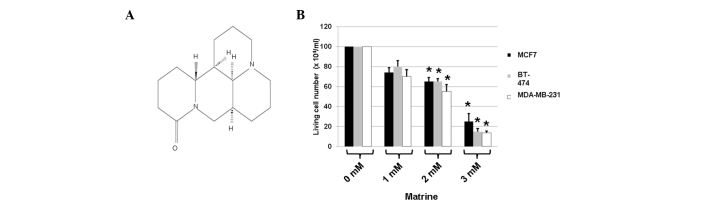
Treatment of cells with matrine. (A) Chemical structure of matrine (chemical formula, C_15_H_24_N_2_O; molecular weight, 248.36). (B) Three breast cancer cell lines (MCF7, BT-474 and MDA-MB-231) were treated with medium only (matrine, 0 mM) or matrine (1, 2 or 3 mM). Cell viability was measured using the 3-(4,5-dimethylthiazol-2-yl)-2,5-diphenyltetrazolium bromide (MTT) assay immediately following 48 h of incubation with matrine. Values are the mean ± SEM for three experiments. ^*^P<0.05 vs. 0 mM matrine.

To determine whether matrine (Fig. 1A) is toxic to breast cancer cell lines, ER-positive MCF7 cells, HER2-positive BT-474 cells and the highly metastatic MDA-MB-231 cell line were treated with medium only (matrine, 0 mM) or matrine (1, 2 or 3 mM). The cell viability was measured using the MTT assay immediately following 48 h of incubation with matrine. The treatment with medium alone served as a control, as the matrine used in the remaining groups was dissolved in medium. The cells were analyzed for differences in cell death following the various matrine treatments by counting the number of living cells in the presence or absence of the aforementioned compounds, using the MTT assay.

The results showed that, in comparison with the untreated cells, the 48 h-treatment with matrine decreased the cell viability of all three types of cancer cells (Fig. 1B). Treatment with 1 mM matrine for 48 h had inhibitory effects on the cell viability of all three types of cells, leading to reductions in such cell numbers (to 71.5–80.2%) compared with the controls (Fig. 1B). Treatment with 2 mM matrine for 48 h had clear inhibitory effects on the cell viability of all three types of cells, leading to reductions in such cell numbers (to 57.6–63.2%) compared with the controls (Fig. 1B). Finally, treatment with 3 mM matrine resulted in reductions in such cell numbers (to 15.5–23.6%) compared with the controls (Fig. 1B). Among the three types of cells, MDA-MB-231 cells were the most sensitive to treatment with matrine (Fig. 1B). These results suggested that matrine exerted significant toxic effects on the breast cancer cells.

### Matrine induces apoptosis in breast cancer cells

As matrine exerted toxic effects on ER-positive MCF7 cells, HER2-positive BT-474 cells and highly metastatic MDA-MB-231 cells, the effects of the compound on apoptosis were determined in all of three types of cells. The cells were treated with medium only (matrine, 0 mM) or matrine (1, 2 or 3 mM) for 48 h. To quantify the apoptotic incidence, a fluorescence microscopic assay was used following staining of the drug-treated cells with Hoescht 33258.

**Figure 2.**
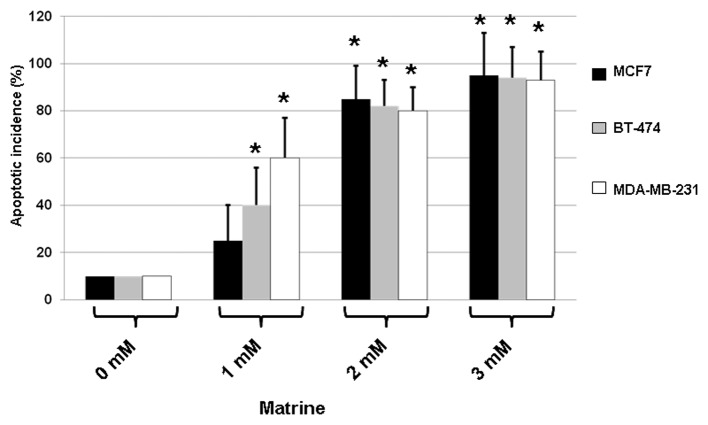
Detection of phenotype-dependent apoptosis induced by treatment with matrine. Three breast cancer cell lines (MCF7, BT-474 and MDA-MB-231) were treated with medium only (matrine, 0 mM) or matrine (1, 2 or 3 mM), and harvested after 48 h. Hoechst 33258-stained cells were examined for apoptotic characteristics (nuclear condensation and chromatin fragmentation) using a fluorescence microscope. The apoptotic incidence was calculated. Data are expressed as the mean ± SEM of three independent experiments. ^*^P<0.05 vs. 0 mM matrine.

As shown in Fig. 2, treatment with matrine resulted in increases in the apoptosis of all three types of cells. When compared with the untreated control, matrine (3 mM) caused the apoptosis of MCF7, BT-474 and MDA-MB-231 cells with incidences of ∼90%. These results indicate that matrine significantly elevated apoptosis in treated cells.

### Matrine treatment leads to the degradation of IKKβ

**Figure 3.**
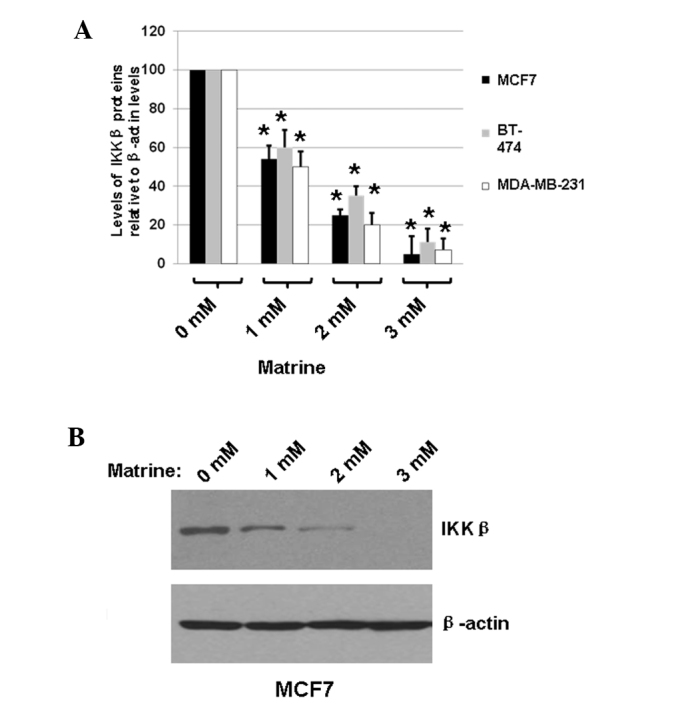
Matrine decreases the expression of the inhibitor of κB (IκB) kinase β (IKKβ). Cells were treated with with medium only (matrine, 0 mM) or matrine (1, 2 or 3 mM), and harvested after 48 h. Whole-cell extracts were prepared and immunoblot analysis was performed to analyze the expression of IKKβ and β-actin. Cellular β-actin served as a loading control. (A) Levels of IKKβ proteins relative to β-actin levels in the MCF7, BT-474 and MDA-MB-231 cells treated with medium only or matrine. The data are from at least three independent experiments. (B) A representative blot using MCF7 cell lysates is shown. Blots using BT-474 and MDA-MB-231 cells are not shown. ^*^P<0.05 vs. 0 mM matrine.

To determine whether matrine inhibited the expression of IKKβ in MCF7, BT-474 and MDA-MB-231 cells, the cells were treated with medium only (matrine, 0 mM) or matrine (1, 2 or 3 mM) for 48 h. The total proteins were extracted and the expression levels of IKKβ were determined using immunoblot analysis, with the cellular β-actin protein serving as a loading control. The mean normalized ODs of the IKKβ protein bands relative to the ODs of the β-actin bands from the same condition were calculated and subjected to statistical analyses. The calculated ratios of the levels of IKKβ proteins relative to the β-actin levels are shown in Fig. 3A. A representative blot is shown in Fig. 3B.

As shown in Fig. 3, treating MCF7, BT-474 and MDA-MB-231 cells with matrine decreased the expression of IKKβ by ≤95%, according to the calculated OD values of the IKKβ bands relative to the β-actin bands. These results indicated that matrine significantly decreased IKKβ expression in the treated breast cancer cells, suggesting that matrine effectively inhibited the proliferation of breast cancer cells by a mechanism associated with the NF-κB signaling pathway.

## Discussion

Matrine has been demonstrated to possess multiple functions, including acting as an analgesic reagent or against infection by pathogenic microorganisms (1–6). It may also be used as an antioxidant, as it promotes cell metabolism and regulates immune activity (7–9). As matrine has therapeutic effects on various solid tumors, including liver, lung, stomach, esophageal, colorectal, cervical and ovarian cancer, as well as malignant lymphoma (11–13), the present study investigated whether matrine has antitumor effects on three breast cancer cell lines, ER-positive MCF7 cells, HER2-positive BT-474 cells and highly metastatic MDA-MB-231 cells.

In the present study, cell viability was measured using the MTT assay immediately following two days of incubation with matrine. Treatment with 1 mM matrine for 48 h exerted inhibitory effects on the cell viability of all three types of cells, leading to 19.8–28.5% reductions in cell numbers. Furthermore, treatment with 3 mM matrine resulted in 76.4–84.5% reductions in cell numbers. Of the three types of cells, MDA-MB-231 cells were the most sensitive to treatment. The results indicated that matrine reduced the cell viability in a concentration-dependent manner. Furthermore, treatment with matrine resulted in apoptosis. Treatment with matrine also resulted in increases in the apoptotic index of all three types of cells. Compared with the untreated control, matrine (3 mM) caused the apoptosis of MCF7, BT-474 and MDA-MB-231 cells with incidences of ∼90%, indicating that matrine significantly increased the levels of apoptosis in the treated cells.

Treatment of MCF7, BT-474 and MDA-MB-231 cells with matrine decreased the expression of IKKβ by ≤95%, according to the calculated OD values of the IKKβ bands relative to the cellular protein β-actin bands. These results indicated that matrine significantly decreased IKKβ expression in the treated breast cancer cells, suggesting that matrine effectively inhibited the proliferation of breast cancer cells by a mechanism associated with IKKβ (24). In conclusion, the present results suggested that matrine may be a promising reagent for treating breast cancer in the future, following further research.
